# Goal Scoring in Soccer: A Polar Coordinate Analysis of Motor Skills Used by Lionel Messi

**DOI:** 10.3389/fpsyg.2016.00806

**Published:** 2016-05-27

**Authors:** Marta Castañer, Daniel Barreira, Oleguer Camerino, M. Teresa Anguera, Albert Canton, Raúl Hileno

**Affiliations:** ^1^Motor Skills Observation Laboratory, National Institute of Physical Education, University of LleidaLleida, Spain; ^2^Faculty of Sport, Centre of Research, Training, Innovation and Intervention in Sport, University of PortoPorto, Portugal; ^3^Social Psychology and Quantitative Psychology, Faculty of Psychology, University of BarcelonaBarcelona, Spain

**Keywords:** soccer, goal scoring, motor skills, polar coordinate analysis, laterality

## Abstract

Soccer research has traditionally focused on technical and tactical aspects of team play, but few studies have analyzed motor skills in individual actions, such as goal scoring. The objective of this study was to investigate how Lionel Messi, one of the world's top soccer players, uses his motor skills and laterality in individual attacking actions resulting in a goal. We analyzed 103 goals scored by Messi between over a decade in three competitions: La Liga (*n* = 74), Copa del Rey (*n* = 8), and the UEFA Champions League (*n* = 21). We used an *ad-hoc* observation instrument (OSMOS-soccer player) comprising 10 criteria and 50 categories; polar coordinate analysis, a powerful data reduction technique, revealed significant associations for body part and orientation, foot contact zone, turn direction, and locomotion. No significant associations were observed for pitch area or interaction with opponents. Our analysis confirms significant associations between different aspects of motor skill use by Messi immediately before scoring, namely use of lower limbs, foot contact zones, turn direction, use of wings, and orientation of body to move toward the goal. Studies of motor skills in soccer could shed light on the qualities that make certain players unique.

## Introduction

Numerous studies in soccer skills have provided evidence that elite soccer players are distinguished by their combination of physical (Reilly, 2003), technical (Bradley et al., 2013), perceptive, cognitive, and decision-making skills (Lee et al., 2007; Nevill et al., 2008; Roca et al., 2013; Sarmento et al., 2014; Furley and Memmert, 2015), with performance being even further enhanced by their ability to anticipate and react to different game situations (Bishop et al., 2013; Wallace and Norton, 2014). In optimal circumstances, such as playing on a team with a style of play that exploits individual qualities (Eys et al., 2007; Carling et al., 2008) and promotes team cohesion (Taylor and Bruner, 2012), players perform better both technically and tactically, thereby contributing to better overall performance and outcomes.

The analysis of numerous performance indicators (James et al., 2004; Tenga et al., 2010), such as contextual factors (Taylor and Bruner, 2012; Barreira et al., 2015), the occurrence of actions on different areas of the pitch (Lago-Ballesteros et al., 2012), and interactions between players (Wallace and Norton, 2014), has permitted a greater understanding of the dynamics of soccer (Duarte et al., 2012). Elite soccer players need to master both physical and technical skills to meet the ever-increasing tactical demands of soccer (Barreira et al., 2015). Furthermore, a greater use of defense strategies and the concentration of players in the midfield have narrowed the performance gap between teams (Wallace and Norton, 2014), facilitating recovery of possession and giving rise to more situations of numerical inferiority in the central path of the pitch (Barreira et al., 2014).

The above factors have all contributed to an increased demand for motor skill versatility among strikers, as players with a mastery of these skills are able to process information faster and react to changing game situations (Lee et al., 2007; Bishop et al., 2013; Memmert et al., 2013; Wallace and Norton, 2014). They are also more likely to become creative agents for the team by generating original responses (Weigelt and Memmert, 2012; Furley and Memmert, 2015). Strikers need to be multi-skilled and acquire techniques and physical qualities specific to their positional roles (Metikoš et al., 2003; Sheppard and Young, 2006). The biomechanics underpinning these multifaceted skills are essential for executing soccer moves, such as, changes of direction (body), axial movements (turns and pivots), and symmetrical and asymmetrical limb actions (Dörge et al., 2002; Velotta et al., 2011). These mechanics of movement are also associated with the effectiveness of actions such as anticipatory moves by kickers and goalkeepers (Weigelt and Memmert, 2012).

Laterality is also relevant in the use of motor skills (Vallortigara, 2006; Loffing et al., 2015). The term *laterality* refers not only to a person's preference for one side of their body (hands, feet, senses, etc.), but also to how they use and orientate their body in space (Castañer et al., 2012). For example, if we want to perform a precision action with our dominant right leg, we will use our left leg to support our body as it turns to the left. Teixeira and Paroli (2000) claim that lateral asymmetries of performance in adults is manifested through a greater use of the dominant limb, particularly for complex motor actions (such as shooting), and that preference for one side of the body over the other appears to be largely determined by use, habit, and confidence. Castañer et al. (2012) used the term *mastering lateral synergy* to refer to an athlete's ability to combine the precision of his/her dominant limb with the stability offered by the other non-dominant limb. Changes in body orientation require stability, particularly in sports such as soccer, which has increased in intensity in recent decades (Di Salvo et al., 2007). Teixeira et al. (2011) noted that stabilization was a fundamental component of performance, as a stable base minimizes variability in execution of movements, and confirmed that compared with non-soccer players, soccer players show a greater preference for use of their non-dominant leg for stability purposes. Several studies have reported that soccer players' left-right preferences can be explained by the functional advantage gained by using their dominant leg (Dörge et al., 2002; Nunome et al., 2006). Thus, laterality allows soccer players to exercise precise control over their movements, which is also an important indicator of performance quality (Teixeira et al., 2011). Kicking direction is also influenced by laterality, and is one of the factors that has been evaluated in studies of goal-side selection in penalty kicking (Weigelt and Memmert, 2012).

The study of motor skills in elite players is still largely based on subjective judgments (see e.g., Duch et al., 2010), but given the importance of these skills, we believe they are worthy of objective, scientific analysis. Lionel Messi, for example, is considered to be a highly skilled player. Regarded as one of the world's best-ever players, he holds numerous goal records and individual awards, including five FIFA Ballons d'Or, three European Golden Shoes, and FIFA World Player of the Year. Soccer experts, such as managers, coaches, and athletic trainers, largely attribute Messi's extraordinary achievements to his motor skills, i.e., to skills built on complex intentional actions (Fry et al., 2014; Murgia et al., 2014; Schaefer, 2014). Nonetheless, these expert opinions are largely based on subjective judgment. The use of motor skills in each soccer action (ball control, dribbling, feints, volleys, shots) joint to lower limb dominance (McGrath et al., 2015) enables players to create opportunities and respond effectively to the task-specific (Velotta et al., 2011) of the game. These aspects, however, remain to be studied through an objective lens, and the current dynamics of soccer calls for research into how elite players use specific motor skills (Murgia et al., 2014) and, in particular, into which patterns of use result in successful outcomes (e.g., goals). The aim of this study was to objectively analyze how, independently of the tactical context of the match, Messi uses his motor skills to resolve game situations just after receiving a pass and just before scoring a goal

## Methods

We chose an observational methodology design for the purpose of our study, as this method has proven effective in the analysis of attacking play in soccer (Jonsson et al., 2006; Camerino et al., 2012b; Lapresa et al., 2013) and of motor skills in physical activity and sport (Castañer et al., 2009; Camerino et al., 2012a). We employed an I/S/M design (Blanco-Villaseñor et al., 2003), where I refers to *idiographic* (focusing on a single player, Messi), S refers to *intersessional* follow-up (recording of numerous matches) and *intrasessional* follow-up (continuous recording of specific moves), and M refers to *multidimensional* (analysis of multiple criteria, or levels of response, using a purpose-designed observation instrument).

## Participants

One hundred and three goals scored by Messi were analyzed; each goal sequence was observed from the moment Messi last received a pass to the moment he took a shot that resulted in a goal. The sequences were analyzed using video footage from public television broadcasts. The 103 goals were scored over a decade (2004–2014) in the UEFA Champions League (*n* = 21), the Spanish premier league *La Liga* (*n* = 74), and *Copa del Rey* (*n* = 8). The goal inclusion criteria were a) clear observability of each sequence (Anguera, 2003) and (b) availability of at least two recordings of each sequence from a different angle. The exclusion criteria were (a) recordings that, while clear, did not allow for coding of the sequences and (b) goals scored directly following receipt of the ball. The ethical requirements of observational methodology were applied to the current study and evaluated positively by the ethics committee and therefore performed in accordance with the ethical standards laid down in the 1964 Declaration of Helsinki.

## Materials

The goal sequences were observed using a observation instrument called OSMOS-soccer player, which includes motor skills–related criteria from the OSMOS instrument (Motor Skills Observation System) (Castañer et al., 2009) and contextual and technical criteria from the SOF5 instrument (Football Observational System) (Camerino et al., 2012b). OSMOS-soccer player contains 10 exhaustive category systems (criteria) and 50 mutually exclusive categories distributed within each criterion (Table 1). The first 7 criteria relate to motor skills, while the last 3 relate to the pitch surface and number of defenders Messi beats before scoring.

**Table 1:** **OSMOS-soccer player**.

**Criterion**	**Category**	**Code**	**Description**
Body part	Left foot	LF	Player touches the ball with left foot
	Right foot	RF	Player touches the ball with right foot
	Left leg	LL	Player touches the ball with left leg (not including foot)
	Right leg	RL	Player touches the ball with right leg (not including foot)
	Chest	CH	Player touches the ball with chest
	Back	BA	Player touches the ball with back
	Head	HD	Player touches the ball with head
Foot contact zone	Tip	TI	Player touches the ball with tip of foot
	Outside	OU	Player touches the ball with outside of foot
	Inside	ID	Player touches the ball with inside of foot
	Heel	HL	Player touches the ball with heel
	Sole	SO	Player touches the ball with sole
	Instep	IT	Player touches the ball with instep
	Non-observable	NON	No clear contact zone between player and ball
Body orientation	Facing goal	FG	Player's chest facing rival goal line
	Facing right	OR	Player's chest facing right side line
	Back to goal	BT	Player's back facing rival goal line
	Facing left	OL	Player's chest facing left side line
Turn direction	Right turn	RT	Player makes a full or half turn to the right (vertical axis)
	Left turn	LT	Player makes a full or half turn to the left (vertical axis)
Pivot foot	Right foot pivot	RFP	Player pivots to the right on right foot
	Left foot pivot	LFP	Player pivots to the left on left foot
Locomotion	One Two Three Four Five More	ONE TWO THR FOU FIV MOR	Player takes one step without touching the ball Player takes two steps without touching the ball Player takes three steps without touching the ball Player takes four steps without touching the ball Player takes five steps without touching the ball Player takes more than five steps without touching the ball
Action	Control	CT	Player gains control of the ball following diverse actions
	Dribbling	CD	Player dribbles the ball
	Shot	SH	Player shoots
	Feint (shot) Feint (pass) Feint (change of dir)	SHF PAF DIF	Player pretends to shoot Player pretends to pass Player tricks a defender by changing direction
	Volley	VO	Player makes contact with the ball before it touches the ground
Number of opponents	Zero	ZE	Player advances with no opposition
	One	ON	Player passes one defender
	Two	TW	Player passes two defenders
	Three	TH	Player passes three defenders
	Four	FO	Player passes four defenders
	Five	FI	Player passes five defenders
	More	MO	Player passes more than five defenders
Side	Right wing	RW	Part of the pitch between the right side line and the right midfield
	Right midfield	CR	Part of the pitch between the left midfield and the right side line
	Left midfield	CL	Part of the pitch between the right midfield and the left side line
	Left wing	LW	Part of the pitch between the left side line and the left midfield
Zone	Ultraoffensive 1	UOO	Zone between the goal line and the front of the goal area
	Ultraoffensive 2	UOT	Zone between the front of the goal area and the penalty box
	Offensive	OFF	Zone between the front of the penalty box and the half-way line (excl. center circle)
	Central	CEN	Center circle

Observation system for motor skills in soccer.

**Figure 1:**
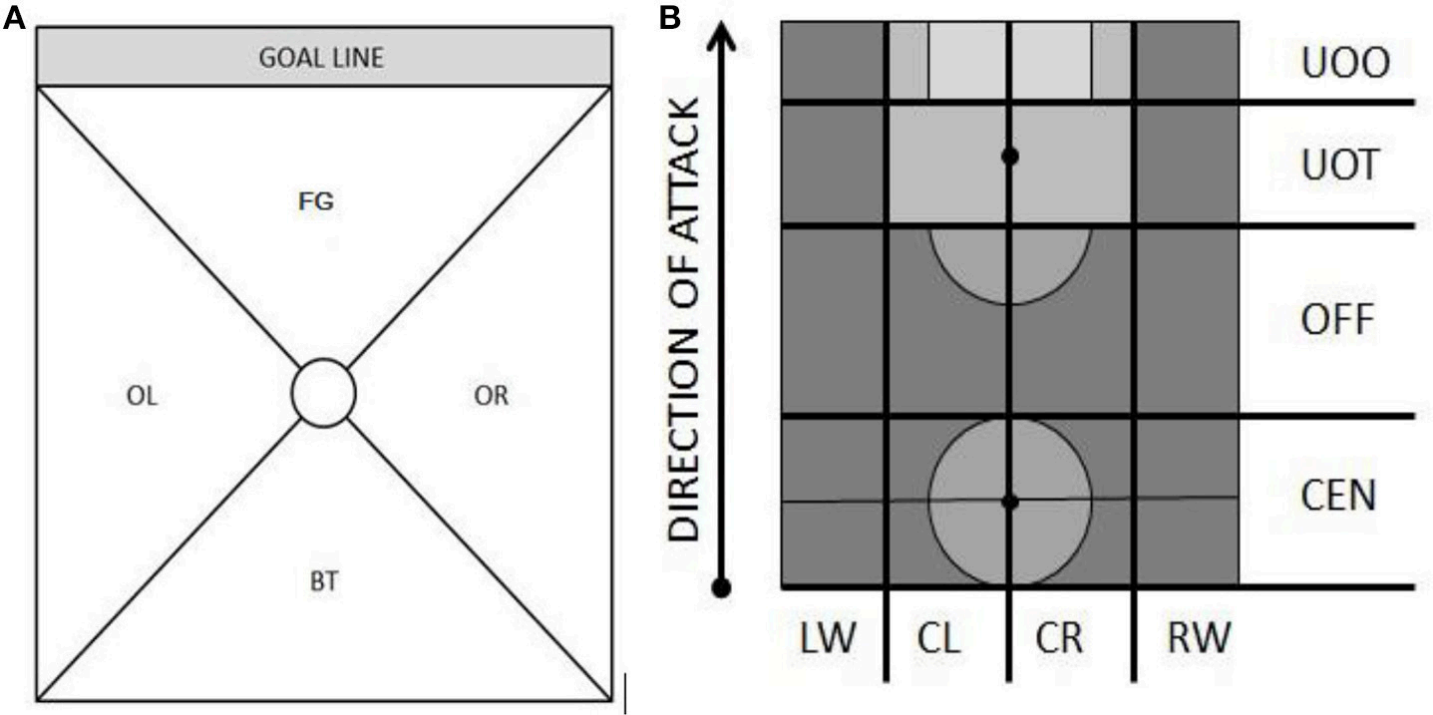
**(A) Body orientation angles, with the center circle representing the player's body**. FG indicates facing goal; BT, back to goal; OL, facing left side line; OR, facing right side line. **(B)** Division of pitch into zones. LW, indicates left wing; CL, left midfield; CR, right midfield; RW, right wing: UOO, ultraoffensive zone 1; UOT, ultraoffensive zone 2; OFF, offensive zone; CEN, central zone.

The Body Part criterion refers to the part of the player's body that comes in contact with the ball. The Foot Contact Zone criterion is recorded as Tip (TI) when the player touches the ball with his toe and as Outside (OU) or Inside (IN) when he touches it with the outer or inner surface of his foot. Body Orientation refers to the angle of the player's body with respect to the goal line when he has control of the ball. There are four categories: facing goal (FR), back to goal (BT), facing right (OR), and facing left (OL; Figure 1A). Turn Direction refers to the direction in which the player turns, while Pivot Foot refers to the foot the player keeps on the ground while turning on one leg. The Locomotion criterion refers to the number of steps the player takes between each touch of the ball. The Action criterion refers to common technical actions, such as control, dribbling, and feints. Number of Opponents refers to the number of defenders that the striker passes during the sequence analyzed (in our case, from the moment Messi received the last pass to the moment he scored a goal). Finally, Zone (criterion adapted from the SOF5 observation system; Jonsson et al., 2006; Camerino et al., 2012b) and Side are used to position the player on the pitch (see Figure 1B for zones). When the player is positioned between two zones on touching the ball, the zone to which he is moving is recorded.

## Procedure

Each goal sequence was analyzed from the moment Messi received the last pass to the moment he shot the ball and scored. The sequences were analyzed by an expert in soccer (a national coach) and an expert in motor skills analysis (a professor from our university), following appropriate training in the use of OSMOS-soccer player using 35 video recordings of goals not included in the sample. Considering that the goal sequences analyzed contained no more than 10 actions and we had two clear recordings of each sequence from different angles, we believe that intraobserver reliability was guaranteed. The recording instrument was LINCE (Gabín et al., 2012) and kappa value for interobserver reliability for the training set (analyzed with LINCE) was 0.92.

Three additional programs were used: (a) GSEQ v5.1.15 (Bakeman and Quera, 2011) to perform a preliminary exploratory analysis of the data, which, consistent with the multidimensional nature of the study were type II data (concurrent, event-based data) (Bakeman and Quera, 1992); (b) HOISAN v1.3.6.1 (Hernández-Mendo et al., 2014), used to perform lag sequential analysis of behaviors with significant associations detected by GSEQ, followed by polar coordinate analysis; and (c) STATGRAPHICS Centurion, v16, a general statistical software package we used to compare proportions.

## Data analysis

Given the large number of codes in the observation instrument, exploratory lag sequential analysis was performed using the multievent data feature in GSEQ v5.1.15 (Bakeman and Quera, 2011; Lapresa et al., 2013) to select categories with significant associations; we included a sufficient number of positive lags (+1 to +5) and negative lags (−1 to −5) to ensure the selection of codes known to be significantly associated (Table 2).

**Table 2:** **Given and conditional behaviors**.

LF	(Body part: left foot)
RF	(Body part: right foot)
RL	(Body part: right leg)
CH	(Body part: chest)
HD	(Body part: head)
OU	(Foot contact zone: outside)
ID	(Foot contact zone: inside)
NON	(Foot contact zone: non-observable)
FG	(Body orientation: facing goal)
OR	(Body orientation: facing right)
BT	(Body orientation: back to goal)
OL	(Body orientation: facing left)
RT	(Turn direction: right)
MOR	(Locomotion: more than five steps)

**Figure 2:**
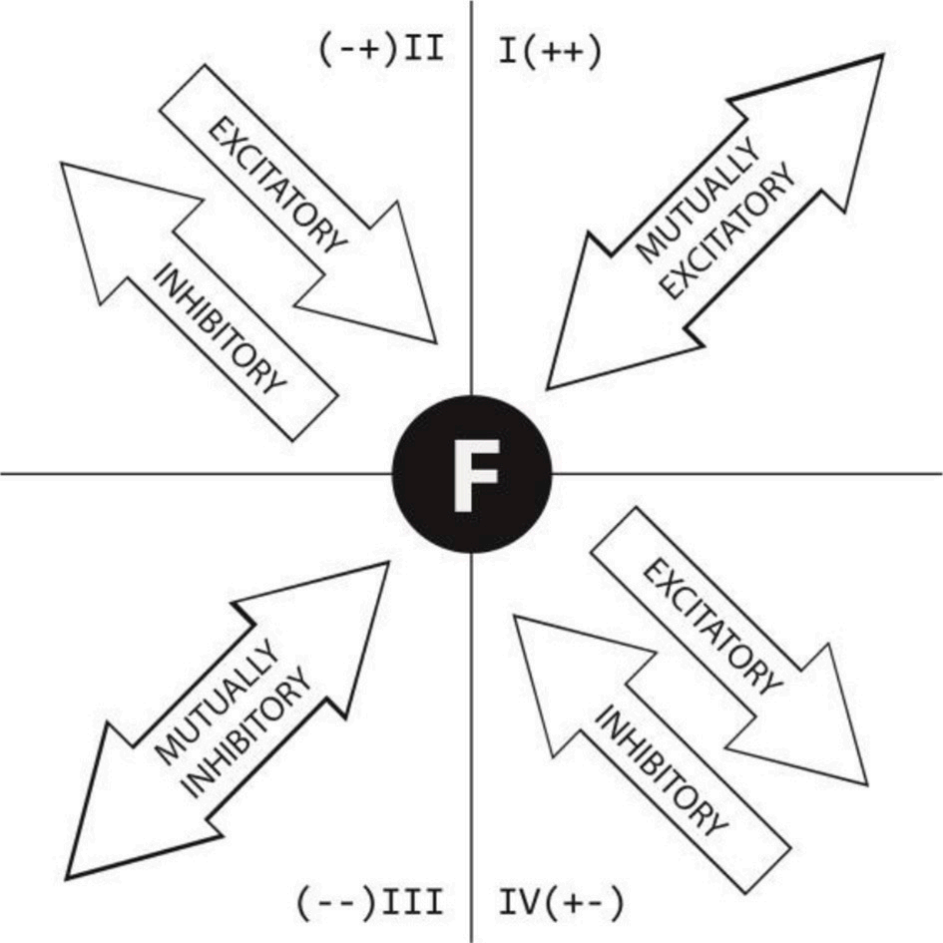
**Graphic depiction of relationships between conditional and given behaviors in polar coordinate maps according to quadrant in which vector is located**.

HOISAN was subsequently used to perform lag sequential analysis of the given and conditional behaviors shown in Table 2. The adjusted residual values obtained in the lag sequential analysis (excitatory and inhibitory actions) were then subjected to polar coordinate analysis (Sackett, 1980). Both prospective (0 to +5) and retrospective (0 to −5) lags were considered. The resulting polar coordinate maps show the associations between each focal behavior (as given behaviors are known in polar coordinate analysis) and all the conditional behaviors analyzed (represented as vectors through the Z_sum_ parameter). The interpretation of these associations varies according to the quadrant in which they are located. Figure 2 provides a graphical explanation of how to interpret the associations between given and conditional behaviors depending on the quadrant in which they are located. In brief, the given behavior is shown as focal behavior in the center of each vector map and the conditional behaviors are located in one of four quadrants. Quadrant I indicates that the focal and conditional behaviors are mutually activated; quadrant II indicates that the focal behavior inhibits the conditional behaviors but is also activated by them; quadrant III indicates that the focal and conditional behaviors are mutually inhibited; and quadrant IV indicates that the focal behavior activates the conditional behaviors but is also inhibited by them. STATGRAPHICS Centurion, v16 was used to explore additional associations between significant categories.

## Results

### Polar coordinate analysis

**Figure 3:**
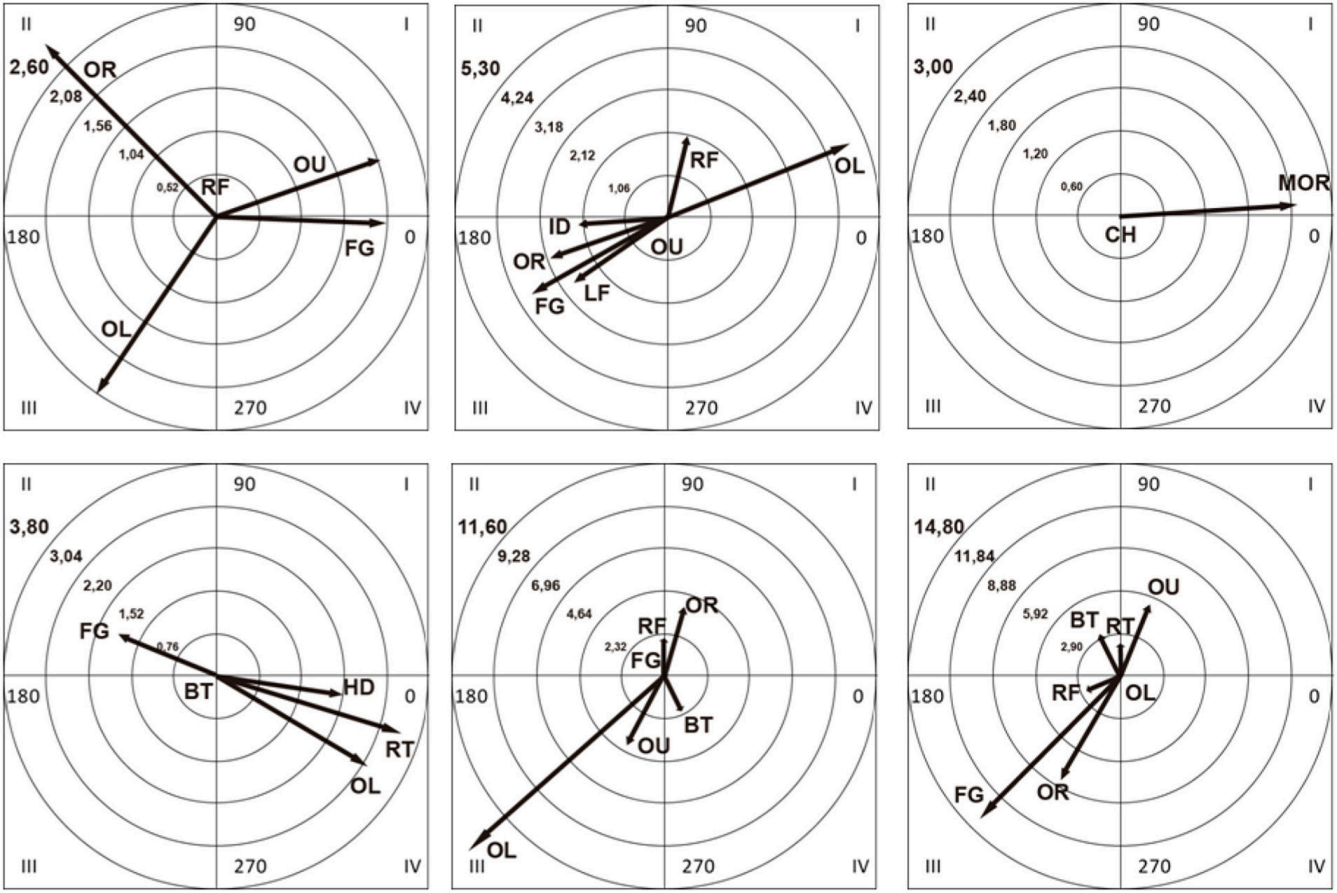
**Polar coordinate maps 1–6 (read from left to right and top to bottom)**.

The polar coordinate maps in Figure 3 show the statistically significant associations (activation or inhibition) between the focal and conditional behaviors. The association is shown both quantitatively (length of vector) and qualitatively (quadrant I, II, III, or IV).

Six polar coordinate maps were obtained (Figure 3). The focal behavior was related to body part use in three cases and *body orientation* in the other three cases.

We will briefly discuss the vectors with a length of >1.96 (*P* < 0.05) representing associations between activations of focal and other behaviors in each map.

In Map 1, there is mutual activation between Messi's use of his right foot (RF) and the outer part of his feet (OU). In addition, this use of his right foot is activated by his facing right (OR) but activates his facing the goal line (FG). Map 2 is directly related to the first map, i.e., Messi's use of his right foot (RF) and the outside of his feet (OU) are mutually activated, but in addition OU activates his facing left (OL). Map 3 shows mutual activation between Messi's use of his chest (CH) and a displacement of more than five steps (MOR). In Map 4, Messi's position when his back is turned to the goal line (BT) is activated by his facing the goal line (FG). As we can see in Map 5, this last position (FG) activates and is activated by his facing right (OR) and is also activated by the use of his right foot (RF), as seen in Map 1. Finally, Map 6 shows mutual activation between Messi's use of his facing left (OL) and the outer part of his feet (OU), which, in turn, is activated by both having his back to the goal line (BT) and turning to the right (RT).

### Comparison of proportions

Table 3 shows the results for the comparison of proportions analysis between pairs of significant behaviors.

**Table 3:** **Comparison of statistically significant proportions between criteria from the OSMOS-soccer player system**.

**Codes**	**Ratio 1**	**Ratio 2**	***P***
LF-RF	266/340 = 0.782	74/340 = 0.217	0.001
OU-ID	83/207 = 0.4	124/207 = 0.599	0.001
OR-OL	35/274 = 0.127	239/274 = 0.872	0.001
ONE-TWO	62/200 = 0.31	18/200 = 0.09	0.001
ONE-THREE	18/200 = 0.09	69/200 = 0.345	0.001
ONE-FOUR	62/200 = 0.31	17/200 = 0.085	0.001
ONE-FIVE	62/200 = 0.31	17/200 = 0.085	0.001
ONE-MORE	62/200 = 0.31	17/200 = 0.085	0.001
RW-CR	20/505 = 0.039	285/505 = 0.564	0.001
CR-CL	285/505 = 0.564	200/505 = 0.396	0.001
UOO-UOT	31/529 = 0.058	292/529 = 0.551	0.001
UOO-OFF	31/529 = 0.058	202/529 = 0.381	0.001
UOT-OFF	292/529 = 0.551	202/529 = 0.381	0.001
UOT-CEN	292/529 = 0.551	4/529 = 0.007	0.001
OFF-CEN	202/529 = 0.381	4/529 = 0.007	0.001

## Discussion

We used polar coordinate analysis, a powerful data reduction technique, to analyze how Lionel Messi uses his motor skills to score goals. We applied the analysis to datasets compiled using a purpose-designed observation instrument, OSMOS-soccer player, comprising 10 broad observational criteria. Seven of the criteria were specifically related to motor skills while the other three were related to external aspects (number of opponents and use of pitch) that might influence actions prior to goal scoring (Jonsson et al., 2006; Lago-Ballesteros et al., 2012; Taylor and Bruner, 2012; Barreira et al., 2014). Our findings show that the number of defenders Messi had to beat before scoring a goal after receiving a pass had no direct influence on goals scored. We believe that this lack of influence is due to the fact that we only analyzed two lags (i.e., events) immediately before a shot at goal. The other nine criteria were significantly associated with goal scoring according to both the polar coordinate and comparison of proportions analyses.

We have structured the rest of the discussion into sections organized by the criteria in OSMOS-soccer player and conclude each section with a discussion of how coaches could incorporate these findings into their practices

### Body contact with the ball

Messi uses his left foot significantly more than his right foot to score goals (*P* < 0.001; Table 3), which is to be expected considering he is left-footed (Fry et al., 2014). Most soccer players are right-footed (Teixeira and Paroli, 2000; Carey et al., 2001; Bloomfield et al., 2007), and use this dominant foot to receive the ball regardless of playing position (Bloomfield et al., 2007). Just a small proportion of soccer players have been found to use both feet with equal effectiveness and skill. Numerous studies have reported that soccer players' left-right preferences can be explained by the functional advantage gained by using their dominant leg. This advantage has been noted for kicking speed (Dörge et al., 2002; Nunome et al., 2006) and complex actions, such as force and direction control in juggling and dribbling, movement timing to trap an approaching ball, and power and accuracy to kick a static or a moving ball (Teixeira and Paroli, 2000; Weigelt and Memmert, 2012). Messi also shows versatility and adaptability in that he uses his right foot when necessary. According to Wallace and Norton (2014), use of the non-preferred foot is a distinguishing feature of elite soccer players that allows them to resolve certain game situations. Coaches should bear in mind that left-footed strikers frequently use their right foot and leg and angle their body to the right to draw defenders away from the left, allowing them to then angle their body to the left and move forward with their left foot, as evidenced in polar coordinate maps 1 and 2.

### Foot contact with the ball

As shown in the first two polar coordinate maps (Figure 3), Messi uses the outside of his right foot while angling his body to the right (map 1). We presume that he may do this to protect the ball from defenders between him and the goal and to create more space and a wider angle to the left. This would explain why the polar coordinate analysis (map 2) showed that Messi's use of his right foot was inhibited by his facing the goal line or the left wing. This observation is further supported by the significant differences detected in the comparison of proportions analysis between use of the outside and inside of the foot (*P* < 0.001) and between angling of the body toward the right and left (*P* < 0.001) (Table 3). Fry et al. (2014) recently reported that Messi benefits from being left-footed. Polar coordinate map 2 shows that the outer and inner part of Messi's feet are mutually inhibited; the vector representing use of the inside of his feet, however, is almost always in quadrant II, indicating that this activates the use of the outer part of this foot. We believe that this shows that coaches should, particularly in situations when players are looking to open up spaces between the defenders to the goal line, use the outer part of their foot to gain more speed and be able to move forward more freely, and that they alternate this with the use of the inner part of the foot between touches of the ball. In other words, at least in the case of Messi, the player uses the inside of his foot to control the ball followed by the outside to move forward.

### Body angle toward goal line

As stated by Castañer et al. (2012, p. 133), “postural support enables stasis and blocks movement, which allows the zone involved in gestural precision to execute the dynamics of the corresponding motor action.” We observed this quality in Messi (map 4), as when he had his back to the goal, he turned on his right leg, leaving his left leg to execute the action with greater precision. Laterality, however, does not only refer to left-right preference (Velotta et al., 2011; McGrath et al., 2015), but also to how players orientate their bodies spatially (Castañer et al., 2012; Loffing et al., 2015). Polar coordinate maps 5 and 6 show how Messi tends to angle his body toward the right with respect to the goal line, frequently moving from right to left to create scoring opportunities. In this respect, we also detected significant differences between use of the right wing and use of the right and left midfield areas (*P* < 0.001; Table 3). We believe that Messi's facing the goal line inhibited his facing left (map 5), because when he would have a narrower shooting angle from the left wing. This mutual inhibition between the left wing and goal line is confirmed in map 6. Coaches could advise left-footed strikers on two crucial aspects regarding body orientation: (a) when facing the goal line, they should immediately angle their body to the right to open up space to use their dominant left leg (map 5); (b) they should angle their body to the left to force defenders to follow them, and then move rapidly to the right, with their body facing the right wing to subsequently create space on the left to take a shot with their left foot. The ultimate aim to make the most of left-footed strikers by ensuring that they become skilled in the use of both legs to dribble, control, and shoot the ball.

### Turn direction and pivot foot

When Messi receives the ball and sees an open space several meters ahead, he uses short touches out of reach of his defender to rapidly work his way through the defense. To do this, he also slightly varies the angle of his body, demonstrating his mastery in zig-zag sprinting, a technique that has been reported as necessary for scoring (Metikoš et al., 2003). Messi's use of left turns and pivots and changes of direction also produced associations in the polar coordinate analysis and provides objective data to support the perception that Messi changes direction frequently. Bloomfield et al. (2007) reported that Premier League players in the 2003–2004 season made a mean of 725 ± 203 turns and pivots per match, highlighting the importance of agility in modern-day soccer, particularly in terms of facilitating changes of direction (558 per match). We obviously observed fewer of movements of this type, as we only analyzed sequences of play immediately preceding goals. Our data thus confirm that turns and pivots (ranging from 180° to 360°) are not common in these situations, but that constant switches between right and left angles are. Coaches should advise strikers that to avoid a defender coming toward them, they should look for other paths, while securing the ball, but that to do this, they do not need to turn their body completely but rather give the sensation that they are going to do this in search of new spaces, thereby tricking the defense and creating a more dynamic attack.

### Locomotion

As soccer actions are not isolated, players who move forward while in possession of the ball need to have a wide set of motor skills that permit them to link these actions and take steps while moving the ball forward. Messi occasionally uses other parts of his body, for example, his chest to control the ball in the air. This movement occured in sequences involving several steps (map 3). Locomotor skills, in particular, are an important skill set in this regard and can help soccer players meet the increasing demands for a combination of tactical, technical, and decision-making skills (Lee et al., 2007; Bishop et al., 2013; Murgia et al., 2014; Wallace and Norton, 2014; Furley and Memmert, 2015), and versatility (Memmert et al., 2013; Roca et al., 2013). Running distance covered by players in possession of the ball has increased over the past 25 years, from 1.1% of total distance (Withers et al., 1982) to 1.2–2.4% (Di Salvo et al., 2007). Our results for Messi are consistent with this increase in movement with the ball. Although Messi preferentially takes one or three steps, all the number of steps he took between each touch of the ball (1–5) were significantly associated with goal scoring (*P* < 0.001) (Table 3). This mastery of locomotor skills allows Messi to achieve considerable speed, while maintaining control and possession of the ball. It should be recalled that Messi is a multidisciplinary player, who has proven particularly effective when placed in the false striker position, in the midfield area. In this respect, we observed that Messi covered considerable distances while in possession of the ball, which is consistent with the report by Bradley et al. (2013) that strikers make fewer passes than defenders and midfielders (22.2 ± 9.6 vs. 30.4 ± 13.4) and are also less successful with these passes (78.1 vs. 78.6%). Coaches should encourage skilled strikers such as Messi to receive the ball and cover distances in the midfield to draw the defenders out of this area and force a high back line.

### Use of pitch sides and zone

Our results confirm that Messi is highly versatile in his use of motor skills and adapts the use of his lower limbs to angle his body with respect to the goal line. To do this, he obviously also needs to be able to take quick, versatile decisions, generating original responses (Furley and Memmert, 2015). This ability is supported by our observation that he tends to occupy the right midfield and right wing more often than the other parts of the pitch (*P* < 0.001) (Table 3) as he moves toward the goal, as this would logically afford him a better angle from which to shoot with his left foot. Messi's case is particularly interesting because even though he is left-footed, our analysis confirms he tends to move to the right wing and the midfield to give him room to create. This brings us to a basic yet crucial aspect for coaches, i.e., the importance of positioning other attackers so that they do not occupy the spaces in which a skilled striker is particularly effective. Coaches should also encourage strikers with similar skills to Messi to move toward the offensive zone to draw a foul or take advantage of a defensive slip-up to move into the ultraoffensive zone (Figure 1B), where they will have more opportunities to score or assist in a goal. Such players are particularly valuable in counterattacks.

In sum, Messi's success may in part be due to the fact that defenders are less used to marking left-footed players and strikers pose a triple threat in that they can pass, dribble past opponents, and shoot. Understanding, from a more objective perspective, how players use their motor skills in sequences of actions immediately preceding a goal is crucial for two profiles of coaches: (a) coaches of teams who have to defend a highly skilled striker, as they will be able to better advise their defenders on positioning, and (b) coaches of players with motor skill profiles (Castañer et al., 2009) similar to those of Messi, as they will be able to guide them on how best to angle and use different parts of their body, exploit the necessary areas of the pitch, and throw the defense off balance.

## Conclusions and future lines of study

The aim of this study was to analyze how Messi uses motor skills to resolve game situations just before scoring a goal, regardless of the tactical context of the match. In the discussion section, we have indicated which aspects of our study may be of use to coaches. There are also learning opportunities for sports scientists, as we have shown that an observational methodology such as the one we employed, which includes powerful techniques such as polar coordinate analysis, could provide objective data to complement subjective judgments of motor skill use. Future studies could investigate the use of motor skills by different players in different game situations. It would be particularly interesting to measure the speed with which different actions occur. We did not do this, but we did (subjectively) notice that part of Messi's “magic” appears to be related to his changes of speed, with alternations between fast and slow movements when turning or angling his body to slip his defenders. Studies focusing on motor skill management could provide interesting and complementary insights into technical and tactical aspects of soccer.

## Author contributions

MC developed the project, supervised the design of the study and the drafting of the manuscript. DB was responsible for the review of the literature and the drafting of the manuscript. OC was responsible for the data collection/handling and revised the content critically. MA performed the polar coordinate analysis and the method section. AC and RH collected and analyzed the data and supervised the drafting of the manuscript. All authors approved the final, submitted version of the manuscript.

## Funding

We gratefully acknowledge the support of two Spanish government projects (Ministerio de Economía y Competitividad): (1) La actividad física y el deporte como potenciadores del estilo de vida saludable: Evaluación del comportamiento deportivo desde metodologías no intrusivas [Grant number DEP2015-66069-P]; (2) Avances metodológicos y tecnológicos en el estudio observacional del comportamiento deportivo [PSI2015-71947-REDP]; and the support of the Generalitat de Catalunya Research Group, GRUP DE RECERCA I INNOVACIÓ EN DISSENYS (GRID). Tecnología i aplicació multimedia i digital als dissenys observacionals [Grant number 2014 SGR 971].

### Conflict of interest statement

The authors declare that the research was conducted in the absence of any commercial or financial relationships that could be construed as a potential conflict of interest. The reviewer EC and handling Editor declared their shared affiliation, and the handling Editor states that the process nevertheless met the standards of a fair and objective review.
